# Clinical and radiographic outcomes of cervical disc arthroplasty with Prestige-LP Disc: a minimum 6-year follow-up study

**DOI:** 10.1186/s12891-018-2201-9

**Published:** 2018-08-07

**Authors:** Junfeng Zeng, Hao Liu, Xin Rong, Beiyu Wang, Yi Yang, Xinlin Gao, Tingkui Wu, Ying Hong

**Affiliations:** 1 Department of Orthopedics, West China Hospital, Sichuan University, 37 Guoxue Lane, Chengdu, 610041 Sichuan China; 2 Department of Operation Room, West China Hospital, Sichuan University, Chengdu, 610041 Sichuan China

**Keywords:** Cervical disc arthroplasty, Prestige-LP Disc, Cervical degenerative disc disease, Heterotopic ossification, Adjacent segment degeneration

## Abstract

**Background:**

Cervical disc arthroplasty (CDA) has been considered as an alternative to cervical arthrodesis in the treatment of cervical degenerative disc diseases (CDDD). The aim of this study was to assess the long-term clinical and radiographic outcomes of CDA with Prestige-LP Disc.

**Methods:**

A total of 61 patients who underwent single- or two-level CDA with Prestige-LP Disc were retrospectively investigated at a minimum of 6-year follow-up. Clinical assessments included visual analogue scale (VAS) for neck and arm pain, Neck Disability Index (NDI), and Japanese Orthopedic Association (JOA) score. Radiological evaluations included range of motion (ROM) of the index and adjacent levels, segmental angle, cervical sagittal alignment, heterotopic ossification (HO) and adjacent segment degeneration (ASD).

**Results:**

Significant and maintained improvement in VAS for neck and arm, NDI and JOA were observed after a mean follow-up of 82.3 months (*p* < 0.001). The preoperative ROM of the index level was 9.7°, which was maintained at 2-and 4-year follow-up (9.3°, *p* = 0.597; 9.0°, *p* = 0.297), but was decreased to 8.0° at final follow-up (*p* = 0.019). Mobility was maintained in 80.5% (62/77) of the implanted prostheses at final follow-up. ROM of the superior and inferior adjacent segments, cervical sagittal alignment and cervical angel were all maintained. The incidence of HO was 42.9% at final follow-up, but it did not influence the clinical outcome. Radiographic ASD were detected in 29.5% of the patients. However, the incidence of symptomatic ASD was only 6.6%.

**Conclusion:**

Cervical disc arthroplasty with Prestige-LP Disc demonstrated a maintained and satisfactory clinical outcome at a minimal of 6-year follow-up, with majority of the prostheses remained mobile. Cervical disc arthroplasty with Prestige-LP Dis can be considered as an effective surgical method in treating CDDD.

## Background

Anterior cervical discectomy and fusion (ACDF) has been considered as golden standard surgical procedure in the treatment of cervical degenerative disc disease (CDDD). However, biomechanical study suggested that fusion of the operated level may increase the stress at the adjacent level [1], and accelerate the degeneration of adjacent segment. In the 10-year postoperative follow-up study, Hilibrand et al. [2] reported that the incidence of symptomatic adjacent segment degeneration (ASD) was 2.9% per year after the cervical fusion surgery, and 25.6% of the patients developed symptomatic ASD within 10 years postoperatively.

Cervical disc arthroplasty (CDA) has been established as an alternative to ACDF for treating CDDD over the past decade. Previous studies have demonstrated that CDA achieved equivalent clinical outcome compared with ACDF [3–8]. Cervical disc arthroplasty was developed to maintain motion at the operated segment and theoretically slow down or avoid the occurrence of ASD. However, long-term clinical results and functional sustainability still need to be proven. Moreover, heterotopic ossification (HO) was reported to increase with the follow-up time [9], which may affect the mobility of the device.

Prestige-LP Disc (Medtronic, Memphis, TN, USA) was one of the artificial cervical discs approved by the Food and Drug Administration (FDA) for treating single- and two-level CDDD. The short- and mid-term results of Prestige LP Disc were satisfactory in previous studies [10–12]. To date, long-term clinical and radiographic follow-up results of Prestige-LP Disc were seldom reported, except for two FDA trails [5, 6]. The purpose of this study was to evaluate the clinical and radiographic outcomes of CDA with Prestige-LP Disc in treating single- and two-level CDDD at minimum 6-year follow-up in a single center.

## Methods

### Study design

The retrospective study was approved by the Ethical Committee of West China Hospital of Sichuan University, and informed consent was obtained from all of the patients. There were 78 consecutive patients underwent single- or two- level CDA with Prestige-LP Disc for the treatment of CDDD between January 2008 and July 2011 in our institution. A total of 61 patients who had completed at least 6-years follow-up were included in this study. The other 17 patients were excluded for incomplete data or lost to follow-up. Clinical and radiographic data were routinely collected preoperatively, postoperatively at 1 week and 3, 6, 12, 24, months, and biennially up to minimum of 72 months.

The inclusion criterion was patients with single- or two-level CDDD between C3 to C7 causing radiculopathy or myelopathy that did not respond to at least 6 weeks of non-operative treatment. Exclusion criteria for this study included: radiographic signs of cervical instability or severe facet joint degeneration, ossification of the posterior longitudinal ligament, prior cervical spine surgery, osteoporosis (T-score ≤ − 2.5), ankylosing spondylitis, rheumatoid arthritis, tumor, trauma, infection, and metabolic bone diseases.

### Prosthesis description

The Prestige-LP cervical disc is an unconstrained ball-in-trough articulation composed of titanium ceramic composite. This prosthesis serves to maintain segmental cervical motion and disc space height. The metal-on-metal prosthesis contains dual serrated kneels which are attached to vertebral bodies through impaction for fixation. The prosthesis has various combinations of depth and height for accommodating the intervertebral disc space.

### Surgical procedure

All surgeries were performed by a single senior surgeon using a standard Smith-Robinson approach. A right side transverse skin incision was made at the index level. After thorough exposure, the anterior longitudinal ligament and diseased disc were completely removed, along with the posterior longitudinal ligament and osteophytes if present. After the discectomy and decompression was completed, a high-speed burr was used to carefully prepare the endplate in a flat and parallel fashion. A sized Implant Trial was used to confirm the size of the prepared disc space. Rail Cutter Guide and Bit were used to drill the fixation channels in the endplate. Prestige-LP Disc corresponding to the trial was inserted into the vertebral body. The same procedure was performed at the other level in patient with two-level CDDD. Lastly, lateral and anterior-posterior fluoroscopies were taken to ensure proper placement.

### Outcome assessment

Clinical outcomes were assessed by visual analogue scale (VAS), Neck Disability Index (NDI), and Japanese Orthopedic Association (JOA) score. The VAS scores were used to evaluate the neck and arm pain. The NDI scores were used to assess the function of neck. The JOA scores were used to assess the neurological status.

Radiological examinations consisted of anteroposterior and lateral radiographs, as well as dynamic lateral radiographs. Range of motion (ROM) of the index and adjacent levels were determined on the dynamic lateral radiographs at maximum flexion and extension by measuring the disc space angle. An ROM of less than 2° was defined as failure to maintain the mobility of prosthesis [9]. Segmental angle was defined as the Cob angle of the index level which was measured on the lateral radiograph. Cervical sagittal alignment was measured by the C2–7 angle. The grade of HO was assessed according to McAfee classification [13]. Radiological evidence of ASD was defined on the lateral radiograph by any presence of the following findings: (1) new or enlarged ossification of the anterior longitudinal ligament; (2) a new or increased narrowing of the disc space > 30%; and (3) new anterior enlarged osteophyte formation [14, 15]. The radiographic assessments were conducted by two independent orthopedic surgeons.

### Statistical analysis

Statistical analysis was conducted using SPSS 22.0 (SPSS Inc., Chicago, Illinois, USA). The two-tailed paired t test was used to compare pre- and postoperative results. Results between independent groups were compared using Mann-Whitney U test. Statistical significance is defined as *p* < 0.05.

## Results

### Patient characteristics

This study included 61 patients with a mean follow-up of 82.3 months (range, 72–108 months). There were 28 male and 33 female patients, with a mean age of 44.1 years (range, 26–62 years). A single-level CDA was performed in 45 cases and two-level CDA was performed in 16 cases. A total of 77 Prestige-LP Discs were implanted from C3/4 to C6/7 as demonstrated in Table 1.

**Table 1:** Characteristics of patients

Characteristics	
No. of patients	61
Gender	
Male	28 (45.9%)
Female	33 (54.1%)
Age (years)	44.1 ± 6.7
Follow-up (months)	82.3 ± 9.6
Diagnosis	
Radiculopathy	31 (50.8%)
Myelopathy	17 (27.9%)
Radiculopathy & Myelopathy	13 (21.3%)
Single-level surgery	45 (73.8%)
Two-level surgery	16 (26.2%)
Level of surgery	
C3/4	1 (1.3%)
C4/5	13 (16.9%)
C5/6	39 (50.6%)
C6/7	24 (31.2%)
Total number of implants	77

### Clinical outcomes

**Fig. 1:**
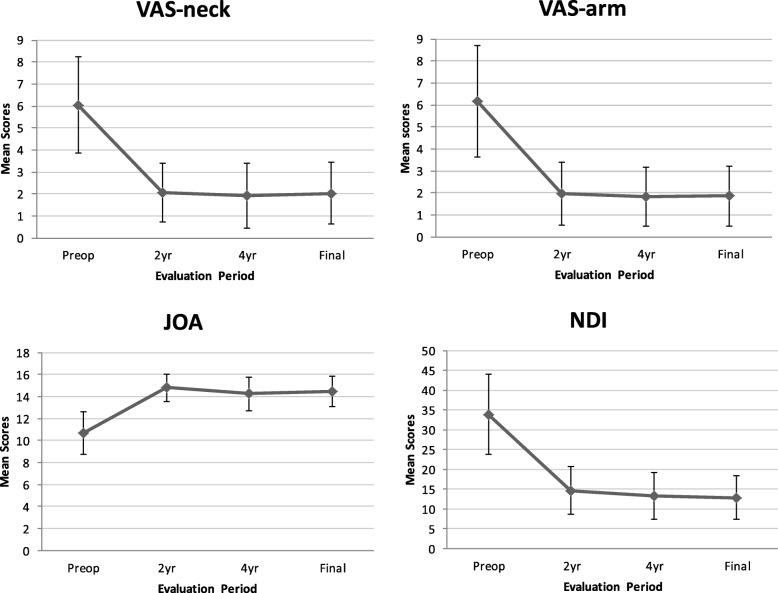
Clinical parameters obtained at different evaluation periods

A statistically significant improvement in VAS, NDI and JOA scores was observed at every evaluation period (Fig. 1). The mean VAS score for neck and arm was significantly decreased from 6.0 ± 2.2 and 6.2 ± 2.5 preoperatively to 2.0 ± 1.4 (*p* < 0.001) and 1.9 ± 1.4 (*p* < 0.001) at final follow-up, respectively. The average preoperative NDI score was 33.9 ± 10.1, which was significantly decreased to 12.9 ± 5.4 (*p* < 0.001) at final follow-up. The NDI scores revealed a mean improvement of 21 points at final follow-up. The overall NDI success rate was 83.6% (at least 15 points improvement based on the FDA criteria). Likewise, the mean JOA score significantly increased from 10.7 ± 1.9 preoperatively to 14.5 ± 1.4 (*p* < 0.001) at final follow-up.

### Radiological outcomes

**Fig. 2:**
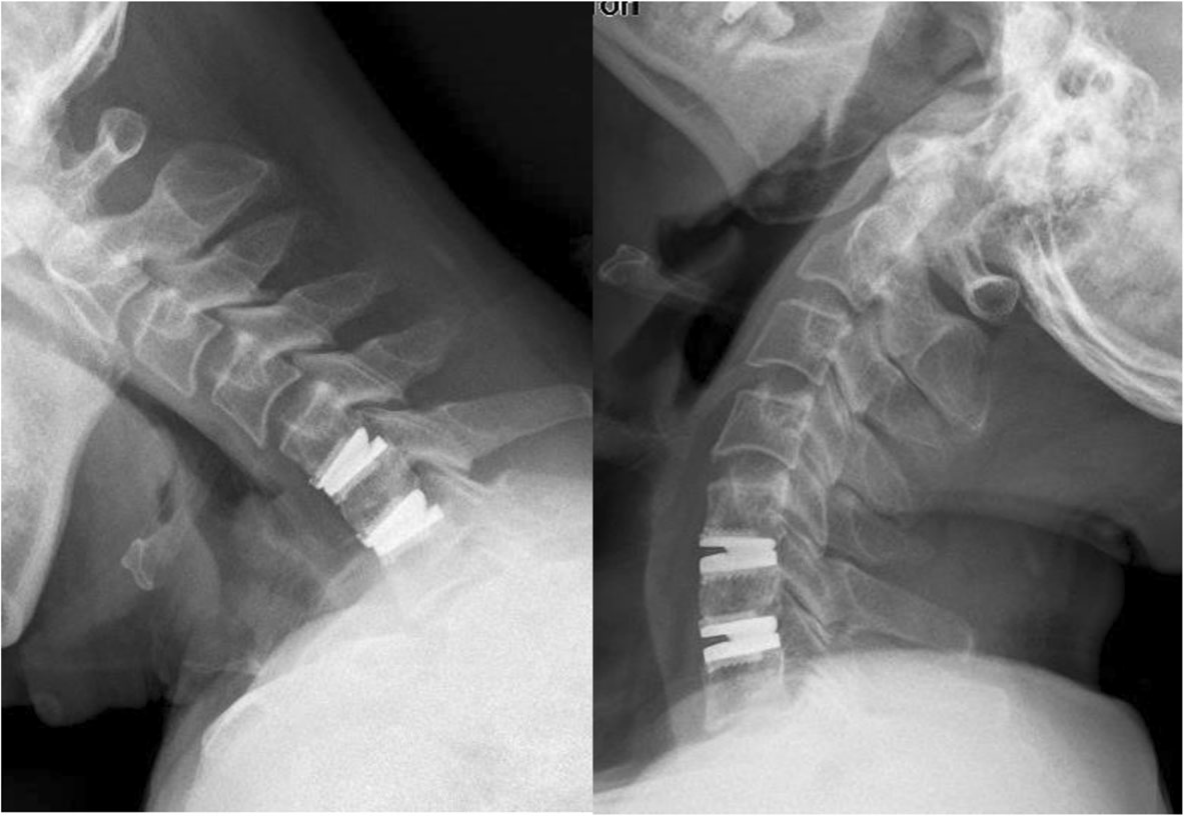
Lateral flexion and extension radiographs showing satisfactory prosthesis mobility at C5/6 and C6/ 7 at 89 months after surgery

Radiological outcomes regarding cervical alignment and ROM are presented in Table 2. The average preoperative cervical sagittal alignment and cervical angle were 10.5 ± 9.3° and 3.1 ± 2.2°, which were maintained at 11.0 ± 9.7°and 2.9 ± 3.6° at final follow-up (*p* = 0.658 and *p* = 0.591), respectively. The mean ROM of the index level was 9.7 ± 4.7° preoperatively and was maintained at 9.3 ± 5.8° and 9.0 ± 5.1° at 2- and 4-year follow-up (*p* = 0.597 and *p* = 0.297), while it was significantly decreased to 8.0 ± 5.6° at final follow-up (*p* = 0.019). Mobility of the prosthesis was maintained in 80.5% (62/77) of the operated segments at final follow-up (Fig. 2). There were no significant differences in ROM of superior and inferior levels between pre-operation and final follow-up (*p* = 0.434 and *p* = 0.463) (Table 2).

**Table 2:** Pre- and post-operative mean cervical alignment and range of motion

	Preoperative	2-year FU	4-year FU	Final FU
Cervical sagittal alignment(°)	10.5 ± 9.3	11.5 ± 9.1	11.4 ± 10.3	11.0 ± 9.7
Segmental angle(°)	3.1 ± 2.2	3.0 ± 3.0	2.8 ± 3.3	2.9 ± 3.6
ROM of operated level(°)	9.7 ± 4.7	9.3 ± 5.8	9.0 ± 5.1	8.0 ± 5.6*
ROM of superior level(°)	10.2 ± 5.1	9.9 ± 5.2	10. 1 ± 5.2	9.5 ± 5.7
ROM of inferior level(°)	10.0 ± 4.3	9.4 ± 3.7	8.8 ± 4.6	9.4 ± 4.7

*FU* follow-up, *ROM* range of motion

*P < 0.05, compared with preoperative

**Fig. 3:**
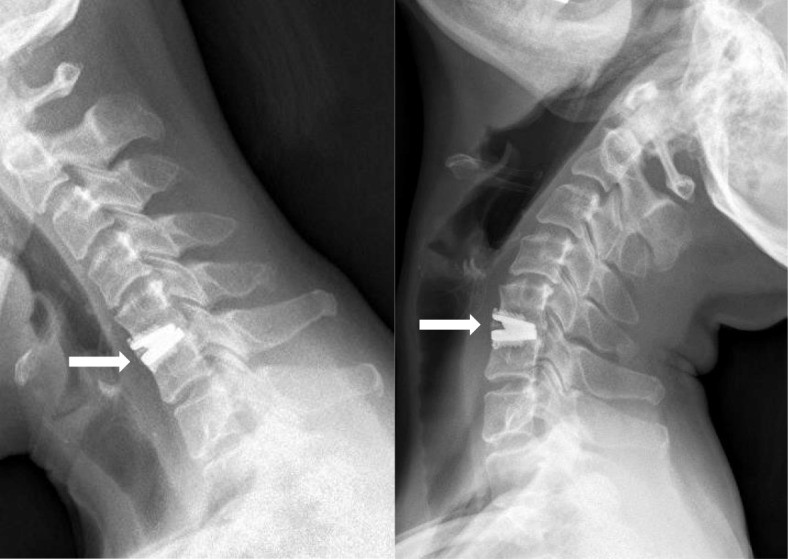
Lateral flexion and extension radiographs showing heterotopic ossification at C5/6 at 72 months after surgery

According to the McAfee classification, the incidence of HO was 23.4% (18/77) and 42.9% (33/77) at 2-year and final follow-up, respectively (Table 3). There were 10 levels (13.0%) with grade 3 HO, and 8 (10.4%) with grade 4 at final follow-up (Fig. 3). The mean ROM for HO group was significant lower than that of non-HO group at final-follow-up (9.5° vs 5.9°, *p* = 0.001). However, no significant differences were seen in VAS for neck and arm, NDI and JOA scores between HO group and non-HO group (*p* = 0.349, *p* = 0.750, *p* = 0.407, and *p* = 0.917).

**Table 3:** Grades of heterotopic ossification at 2-year and final-follow-up

Grade of HO	2-year follow-up	Final follow-up
0	59 (76.6%)	44 (57.1%)
1	6 (7.8%)	7 (9.1%)
2	4 (5.2%)	8 (10.4%)
3	6 (7.8%)	10 (13.0%)
4	2 (2.6%)	8 (10.4%)

In addition, radiological evidence of ASD was observed in 29.5% (18/61) of the patients at final follow-up. The ASD at inferior level was detected in 18 cases, and 3 cases with ASD at superior level. Symptomatic ASD was found in 4 patients (6.6%). Three patients complained neck pain and one patients complained arm pain. All four patients were successfully treated by conservative treatment. No patients required a revision surgery. No prosthesis dislocation or failure was seen in all the 77 implanted prosthesis.

## Discussion

Cervical disc arthroplasty has been accepted as an alternative surgical method for treating CDDD. Previous clinical studies have demonstrated satisfactory short- and mid-term results of CDA with Prestige-LP Disc [10–12]. In our present study, favorable and stable clinical outcome was seen at a minimal of 6-year follow-up. Clinical outcome parameters, including VAS for neck and arm, NDI, and JOA scores, were all significantly improved and maintained at all postoperative evaluation periods compared with those of preoperatively. Similar results were seen in other long-term studies with various types of cervical artificial disc [5–9]. We found an NDI success rate of 83.6%, which was also comparable to the NDI success rate of 86.1% [5] and 87.0% [6] in the two FDA studies. The reported incidence of prosthesis dislocation after CDA varied from 3.1 to 19.6% [9, 16, 17]. No serious adverse events including prosthesis dislocation or failure were occurred in the present study. Our study confirmed that CDA with Prestige-LP Disc can yield satisfactory long-term clinical outcome in treating CDDD.

As cervical disc arthroplasty was designed to preserve motion at the operated level and avoid hypermobility of the adjacent segments, long-term functionality is particularly important. Our study demonstrated that 80.5% of the prosthesis maintained mobile and the mean ROM of the operated level was 8.0° after a mean follow-up of 82.3 months. In addition, cervical sagittal alignment and cervical angle were well maintained. Similarly, Gornet et al. reported a mean operated segmental ROM of 6.78° after single-level Prestige-LP Discs implantation at 84-month follow-up [5]. Lanman et al. reported both the ROM at superior and inferior operated level was above 6° after two-level Prestige-LP disc arthroplasty at 84-month follow-up [6]. Dejaegher et al. reported that 81% of the Bryan cervical disc remained mobile with a mean ROM of 8.6° at 8-year follow-up [7]. In addition, in a 15-year follow-up study of Bryan disc arthroplasty, Pointillart et al. reported that 68.2% (15/22) of the prosthesis maintained mobile with an average of 9° at final follow-up [18]. Previous studies demonstrated that both CDA and ACDF had gained good long-term clinical outcome, and most of the cervical discs remained satisfactory segmental mobility [5, 6, 19]. Furthermore, our study shown maintained ROM at the superior and inferior levels, which means no hypermobility were occurred at adjacent segments. Our data confirmed that Prestige-LP Disc arthroplasty has the potential to maintain long-term mobility at the operated level and avoid hypermobility of adjacent segments.

Heterotopic ossification is well-known occurrence after cervical disc arthroplasty. We noted that 23.4% of the prosthesis developed HO at 2-year follow-up. The incidence of HO was 42.9% at final follow-up. Our study revealed that HO rate was increased with the prolongation of follow-up time. The incidence of HO ranged from 7.7 to 90% at 6–10 years follow-up time with different types of prosthesis in other studies [8, 9, 20, 21]. The progression of HO was also reported in previous studies [8, 9, 22]. According to McAfee classification [13], HO of grade 3 and 4 can damage the ROM of the treated level. We found HO-group had lower ROM than that of non-HO group at final follow-up. However, HO did not influence clinical outcome in the present study. The formation of HO after CDA and its effect on clinical outcome still need further studies.

It is still controversial that ASD is due to cervical fusion or simply the natural degeneration of cervical spine. Kong et al. reported that the prevalence of radiographic ASD following cervical spine surgery was 28.28% in a Meta-analysis [23]. In a 10-year follow-up of asymptomatic volunteers and patients underwent cervical fusion, Matsumoto et al. found that both ACDF patients and healthy subjects shown progression of disc degeneration, but ACDF patients had higher incidence of progression of degeneration at adjacent segments than healthy subjects [24]. Lee et al. investigated the natural history of cervical degeneration and ASD of patient underwent cervical fusion in a systematic review [25]. Similarly, they concluded that ASD may occur at a higher rate than natural cervical degeneration, and biomechanical effect of fusion may accelerate pathologic changes at adjacent segments. Previous biomechanical study also demonstrated that fusion may increase the stress at the adjacent segments, and accelerate its degeneration [1].

Cervical disc arthroplasty aims to maintain the segmental motion and then theoretically reduce or slow down the occurrence of ASD. Lower incidence of ASD were reported in other long-term studies when compared CDA with ACDF [3, 4, 26]. We found a radiographic ASD in 29.5% of the patients at final follow-up. However, only 6.6% of the patients developed symptomatic ASD. Zhao et al. reported the rate of radiographic ASD was 47.6% at 10-year follow-up after Bryan cervical disc arthroplasty [21]. Quan et al. noted 19% of patients had radiographic ASD after 8-year follow-up of Bryan disc [9]. Mehren et al. found 35.7% of the patients developed radiographic ASD at 10-year follow-up of Prodisc C disc [8]. Whether ASD can be reduced by CDA remains to be investigated. Because HO damaged the mobility of cervical disc, the correlation between HO and ASD is of particular importance in future studies.

Our study has some limitations. Firstly, this was a retrospective study and lack of a control group. For this reason, we cannot directly compare the result with ACDF. Secondly, the sample was relatively small compared to the previous FDA studies [5, 6]. However, all of the surgeries in our study were performed by a single senior surgeon in a single center. Lastly, it is challenging to precisely evaluate the degeneration of adjacent segments without postoperative MRI imaging of cervical spine. However, we still can adequately assess ASD according to above mentioned radiographic criterion. Future randomized control trials were needed to further evaluate the functional and clinical results of CDA.

## Conclusion

Cervical disc arthroplasty with Prestige-LP Disc demonstrated maintained and significant improvement in all measured clinical parameters at a minimum 6-year follow-up. Radiological evaluations shown 80.5% of the prostheses maintained mobility with a mean ROM of 8.0°. Though the incidence of HO was 42.9%, HO did not influence the clinical outcome. Hypermobility were not occurred at the adjacent segments and a low incidence of symptomatic ASD was detected. Cervical disc arthroplasty with Prestige-LP Dis can be regarded an effective surgical method in treating CDDD.

## Abbreviations

ACDF: Anterior cervical discectomy and fusion
ASD: Adjacent segment degeneration
CDA: Cervical disc arthroplasty
CDDD: Cervical degenerative disc diseases
HO: Heterotopic ossification
JOA: Japanese Orthopedic Association
NDI: Neck Disability Index
ROM: Range of motion
VAS: Visual analogue scale
